# An etiological reappraisal of pancytopenia - largest series reported to date from a single tertiary care teaching hospital

**DOI:** 10.1186/2052-1839-13-10

**Published:** 2013-11-06

**Authors:** Arvind Jain, Manjiri Naniwadekar

**Affiliations:** 1 Laboratory Medicine Department, Imperial College London Diabetes Centre (ICLDC), P.O. Box no. 222464, Al Ain, United Arab Emirates; 2 Department of Pathology, Krishna Institute of Medical Sciences University (KIMSU), Karad, Maharashtra, India

**Keywords:** Pancytopenia, Hypersplenism, Infections, Myelosuppressants, Megaloblastic anemia, Hypoplastic aplastic anemia, Leukemia, Lymphoma, Plasma cell dyscrasia, Myelodysplastc syndrome

## Abstract

**Background:**

Peripheral pancytopenia is not a disease by itself; rather it describes simultaneous presence of anemia, leucopenia and thrombocytopenia resulting from a number of disease processes. Only a few systematic studies have been published on the topic of pancytopenia, although extensive studies have been done for its different etiological factors like aplastic anemia, megaloblastic anemia, leukemia, etc. Thus, this study was carried out to investigate for and to identify the causes of pancytopenia, to find out the frequency of different causes, to determine the incidence of pancytopenia in relation to sex and age and to compare our findings with those of other similar studies from this part of the world.

**Methods:**

This was a prospective study conducted in the Department of Pathology of a teaching institute and a tertiary care hospital in southern Maharashtra, India, over a period of two years. All the patients referred to the central clinical laboratory for routine complete blood count (CBC) and peripheral smear (PS) examination, from both - the outpatient and the inpatient departments, were screened for pancytopenia. Of these, a total number of 250 cases that fulfilled the diagnostic criteria were selected.

Detailed hematological investigations followed by bone marrow aspiration wherever indicated and possible were performed according to standard methods to ascertain the causes of pancytopenia.

**Results:**

A definite male preponderance was observed, the male to female ratio being 2.6 : 1. The majority of cases were encountered in 3rd and 4th decades. Hypersplenism (29.2%), Infections (25.6%), Myelosuppressants (16.8%) and Megaloblastosis (13.2%) were the four most common causes in this large series on pancytopenia from a single centre in India.

**Conclusion:**

Detailed clinical history and meticulous physical examination along with baseline hematological investigations, provides invaluable information in the complete workup of pancytopenic patients, helping in systematic planning of further investigations to diagnose and ascertain the cause, avoiding a battery of unnecessary tests.

## Background

Peripheral pancytopenia is reduction in all three major formed elements of blood to levels below their lower normal limit leading to simultaneous presence of anemia, leucopenia and thrombocytopenia. Thus, it is not a disease entity by itself, but rather a triad of findings [1].

It is a striking feature of many serious and life threatening illnesses and may be caused by several disorders ranging from simple drug-induced bone marrow hypoplasia and megaloblastic anemia to fatal aplastic anemia and leukemias. The mechanism of development of pancytopenia varies from decrease in hematopoietic cell production as in aplastic anemia, trapping of normal cells in hypertrophied and overactive reticuloendothelial system as in hypersplenism, ineffective hematopoiesis in megaloblastosis or replacement by abnormal or malignant tissue in the marrow [1,2].

Although pancytopenia is a relatively common hematological entity and a serious clinical problem with exhaustive differential diagnoses, there is relatively little discussion on this abnormality in major textbooks of hematology and internal medicine [3].

A look at literature shows that there aren’t many comprehensive studies on this subject from developed world, though extensive studies have been done for its individual etiological factors like aplastic anemia, megaloblastic anemia, leukemia, myelodysplastic syndrome, etc. [4].

As the severity of pancytopenia and the underlying pathology determines the management and prognosis of these patients [4], identifying the correct etiopathology in a given case is crucial and helps in implementing timely and appropriate treatment. Thus, this study was conducted mainly with the twin aims of diagnosing the patients with pancytopenia and finding out the common disease entities responsible for pancytopenia.

## Methods

This prospective study was carried out over a period of two years in the Department of Pathology, of a teaching institute and a tertiary care hospital exclusively catering to the rural population of southern Maharashtra state of India. Approval from local ethical committee and institutional review board of Krishna Institute of Medical Sciences University (KIMSU) was obtained to conduct this study. The institutional ethical committee did not feel that written informed consent was required to conduct this study and thus deemed it to be not applicable/waived.

All the patients referred to the central clinical laboratory of the hospital for routine complete blood count (CBC) and peripheral smear (PS) examination, from the outpatient and the inpatient departments were screened for pancytopenia and a total number of 250 cases were selected, based on the criteria’s defined by deGruchy [1] as follows:

1) Hemoglobin (Hb.) level – below 13.5 g/L for males and below 11.5 g/L for females.

2) Total Leucocyte Count (TLC) - below 4 × 10^9^/L.

3) Platelet (Plt.) count – below 150 × 10^9^/L.

In all patients, a complete relevant medical history including age, sex, smoking status, alcohol intake, history of any treatment, intake of or exposure to potentially toxic chemicals, agents or drugs, radiation exposure, history of symptoms such as bone pains, fever, night sweats, malaise, weight loss and pruritus was taken. A detailed meticulous physical examination of every patient was done for pallor, jaundice, hepatosplenomegaly, lymphadenopathy, sternal tenderness and gum hypertrophy. Evidence of hypersplenism and primary malignancy was searched for whenever necessary. Basic hematological investigations like CBC, reticulocyte count, and PS examination were performed in each case.

Blood counts were done by semiautomated electronic cell counter (Sysmex KX – 21, Transasia Biomedicals) and were again crosschecked manually during PS examination. Buffy coat preparation stained by leishman stain was used wherever the TLC was markedly reduced. Bone marrow aspiration studies using standard methods were done wherever indicated and possible, avoiding the cases where the cause for pancytopenia was obvious. In cases of failed aspiration due to dry / bloody tap, insufficient cells, or hypoplastic marrow, a bone marrow trephine biopsy was done from anterior superior iliac spine using standard methods.

Wherever indicated, other investigations performed included erythrocyte sedimentation rate (ESR), urine and stool examination, liver and renal function tests, serological investigations for enteric fever, blood culture, ELISA for HIV, hepatitis B and C viruses, chest and bone radiographs, abdominal ultrasonography, urinary Bence Jones proteins and serum electrophoresis; the investigative workup being directed by the suspected underlying pathology and the provisional diagnoses.

All the patients thus selected were investigated in a systematic manner, cause of pancytopenia was ascertained and the data was analyzed on the basis of etiology, clinical and hematological findings. Clinico-pathological correlation was done in all cases before reaching a definitive diagnosis.

## Results

**Figure 1:**
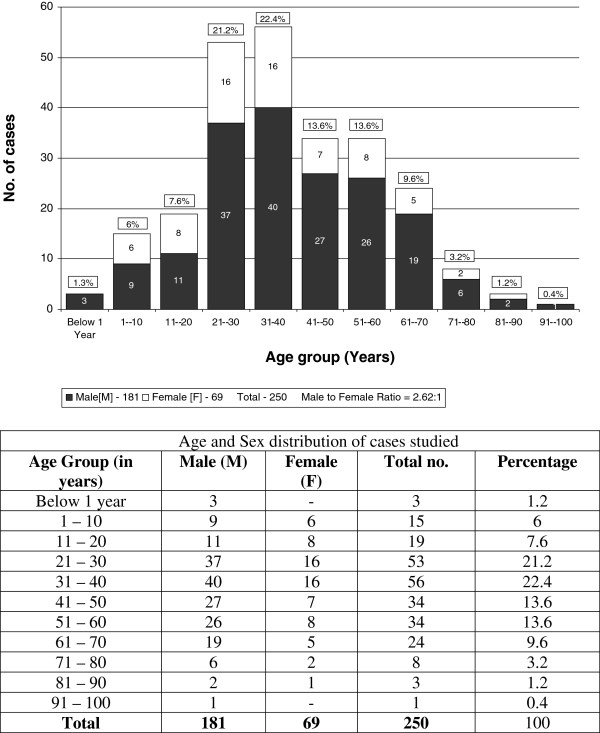
Age and sex distribution of cases studied.

Of the 250 cases studied, 181 (72.4%) were males and 69 (27.6%) females (Figure 1). A definite male preponderance was seen in the overall picture as well as in all age groups, the overall male to female ratio being 2.6: 1. The age range of patients in our study was 2 months to 95 years. The maximum number i.e., 146 (58.4%) cases occurred below the age of 40 years, with majority i.e., 109 (43.6%) cases occurring in 3rd and 4th decades (Figure 1). A total of 56 (22.4%) cases presented between 31 – 40 years of age making it the commonest age group for presentation (Figure 1). In this large series on pancytopenia from a single centre in India, the most common cause was hypersplenism (29.2%) followed by infections (25.6%), myelosuppressants (16.8%) and megaloblastosis (13.2%) (Table 1).

**Table 1:** Etiological breakup of 250 cases of pancytopenia

**Causes**	**No. of cases**
**Hypersplenism**	
Congestive splenomegaly	45
Malaria	15
Tropical splenomegaly	3
Thalassemia	1
Hodgkin’s Disease	1
Idiopathic	8
*Total*	**73 (29.2%)**
**Infections**	
AIDS	30
Septicemia	14
Enteric fever	9
Tuberculosis	8
Viral hepatitis	3
*Total*	**64 (25.6%)**
**Myelosuppressants**	
Carcinoma	18
Leukemia	7
Non-Hodgkin’s lymphoma (NHL)	5
Hodgkin’s disease	2
Others	10
*Total*	**42 (16.8%)**
**Megaloblastosis**	**33 (13.2%)**
**Hypoplastic / Aplastic anemia**	**12 (4.8%)**
**Leukemia**	**7 (2.8%)**
**Lymphoma**	**2 (0.8%)**
**Plasma cell dyscrasia (PCD)**	**2 (0.8%)**
**Myelodysplastic syndrome (MDS)**	**1 (0.4%)**
**Miscellaneous**	**14 (5.6%)**
*Grand Total*	*250 (100*%*)*

Of the 73 (29.2%) cases of hypersplenism, 45 (61.6%) cases were of congestive splenomegaly (Table 1), 36 cases of which had signs of portal hypertension and the remaining nine cases, were of congestive cardiac failure. Of the 36 cases of portal hypertension, 27 were known cases of alcoholic liver cirrhosis, five suffered from hepatitis B infection (HBsAg positive), two cases were diagnosed as having cryptogenic liver cirrhosis and one case each of portal cavernoma and splenic vein thrombosis.

The second commonest cause of hypersplenism in this series was malaria, n = 15 (20.5%) cases (Table 1), of which seven were Plasmodium vivax positive, five were Plasmodium falciparum positive and three had mixed infection, of which one showed signs of disseminated intravascular coagulation (DIC).

In the remaining 13 cases of hypersplenism, eight (10.9%) were labeled as idiopathic, as no cause could be found, three (4.2%) were diagnosed of having tropical splenomegaly, and one case (1.4%) each of thalassemia and Hodgkin’s lymphoma (Table 1).

Infections causing pancytopenia was the 2nd commonest cause of pancytopenia in our study accounting for 64 (25.6%) cases. Known cases of AIDS was the commonest cause accounting for 30 (46.9%) cases followed by patients admitted with septicemia, n = 14 (21.9%) cases. The 30 known cases of AIDS in our study were additionally also suffering either from tuberculosis, cryptococcal meningitis, pneumocystis carinii pneumonia or hepatitis B infection.

Of the 14 cases of septicaemia, seven had gram–negative sepsis (three with Kleibseilla, two with Pseudomonas and two with Escherichia coli) and four had gram-positive sepsis (two with Staphylococci and two with Streptococci). In the remaining three patients no cause could be found for their clinical presentation of septicaemia with pancytopenia. Of these 14 cases, 6 (42.8%) cases succumbed to infection.

We had nine (14%) cases of enteric fever with pancytopenia in this group (Table 1). All of them presented as pyrexia of unknown origin (PUO); their CBC reports showing varying degree of pancytopenia, no particular significant trend being observed. All of them showed significantly positive widal test. One of the patients showed resistance to Quinolones while other two developed complications like acute psychosis and DIC.

Of the eight (12.5%) cases of tuberculosis in this group, three cases were of pulmonary tuberculosis, two of abdominal tuberculosis and one each of tuberculous osteomyelitis, tuberculous meningitis and tuberculosis of chest wall.

We also had 3 patients in this group diagnosed to be suffering from viral hepatitis but tests for hepatitis A, B & C virus were negative.

The third cause for pancytopenia was the use of myelosuppressants accounting for total 42 (16.8%) cases, of which 18 (42.8%) had either carcinoma (Table 1) of breast, oral cavity, gastrointestinal tract, hypopharynx, cervix, choriocarcinoma and were taking regular chemotherapy and or radiotherapy.

Of the seven cases of leukemia on chemotherapeutic agents, four (57%) cases were acute lymphoblastic leukemia (ALL), two were chronic myeloid leukemia (CML) and one case of acute myeloid leukemia (AML) - M4. Also, there were five cases (11.9%) of Non-Hodgkin’s lymphoma (NHL) and two cases (4.8%) of Hodgkin’s lymphoma in this group (Table 1). Antineoplastic agents like Methotrexate, Cyclophosphamide, Vincristine and 5-Flurouracil were used in various combinations as per the regime used and pancytopenia developed during the course of the therapy.

Of the 10 cases in others group, four cases were on chronic use of analgesics for arthritis or spondylosis, two cases were on Dapsone for leprosy, two on Neomercazole for thyrotoxicosis and two on antiepileptics – Eptoin and Carbamazepine each.

Of the 33 (13.2%) cases of megaloblastosis, 18 (54.5%) cases showed pure megaloblastic anemia of varying severity whereas 15 (45.5%) cases showed dimorphic anemia i.e., combination of iron deficiency and megaloblastic anemia in varying proportions, the megaloblastic change being partially masked by the superadded iron deficiency.

Of the 12 (4.8%) cases in hypoplastic / aplastic anemia group, eight cases were of hypoplastic anemia and four of aplastic anemia, as established by bone marrow findings. Of the eight cases of hypoplastic anemia, one case had a history of viral hepatitis a month preceding his pancytopenia and one case was HIV positive. Of the four cases of aplastic anemia, one case had history of chronic alcoholism and one patient had a thymoma removed (operatively). In the remaining 8 (66.7%) cases, no cause for hypoplasia or aplasia could be identified and so were labeled as idiopathic.

Of the seven (2.8%) cases in leukemia group, four cases were ALL and three were AML (two cases of M3 and one of M1).

Of the 14 cases in the miscellaneous group, two were pregnant women, two had a history of passing worms, one was a known case of malabsorption syndrome, one case each was suffering from ulcerative colitis, chronic amoebiasis, pancreatitis and chronic renal failure. All the cases showed a dimorphic blood picture and so the pancytopenia was thought to be due to nutritional deficiency of Iron, Vitamin B_12_ and Folic acid, once again hinting towards the higher prevalence of nutritional anemia’s in Indian subjects especially in rural population. As these patients did not turn up again after their first visit, further investigations to find the cause of pancytopenia could not be done.

## Discussion

The variation in the frequency of various diagnostic entities causing pancytopenia in different population groups (Table 2) has been attributed to differences in methodology and stringency of diagnostic criteria, period of observation, geographic area, age pattern, nutritional status, prevalence of infective disorders, genetic differences, and varying exposure to myelotoxic agents amongst other factors [5].

**Table 2:** Comparison of number of cases, age and sex distribution and four most common causes of pancytopenia in different studies conducted in different countries

**Study**	**Country**	**Year**	**No. of cases**	**Age group**	**M : F ratio**	**Commonest cause**	**2nd common cause**	**3rd common cause**	**4th common cause**
**Kale P et al.**[6]	India	1991	70	All	-	HS (47.6%)	MA (25.4%)	AL (14.5%)	Infections (7.25%)
**Tilak and Jain et al.**[4]	India	1999	77	5 to 70	1.1 : 1	MA (68%)	AA (7.7%)	Other causes (24.3%)	
**Savage et al.**[7]	Zimbabwe	1999	134	All	-	MA	AA	AL	AIDS
**Kumar et al.**[5]	India	2001	166	12 - 73	2.1 : 1	AA (29.5%)	MA (22.3%)	Aleukemic leukemia (12%)	HS (11.4%)
**Iqbal W et al.**[8]	Pakistan	2001	208	All	-	MA (28.3%)	AA (22.1%)	HS (14.4%)	NHL (5.3%)
**Khunger et al.**[9]	India	2002	200	2 - 70	1.2 : 1	MA (72%)	AA (28%)	Subleukemic leukemia (5%)	HS (3%)
**Niazi M. et al.**[10]	Pakistan	2004	89	1 - 75	1.7 : 1	BM Aplasia (38.3%)	MA (24.7%)	HS (18.4%)	AL (13.6%)
**Ishtiaq O et al.**[3]	Pakistan	2004	100	12 - 82	1.1 : 1	MA (39%)	HS (19%)	AA and HA (10%)	MDS (5%)
**Hamid et al. **[11]	Yemen	2008	75	3 - 85	1.03 : 1	HS (45.3%)	MA (14.7%)	AA (13.3%)	AL (13.3%)
**P. M. devi et al.**[12]	India	2008	50	3 - 80	1.5 : 1	HA (22%)	MA (18%)	MDS (18%)	Subleukemic leukemia (14%)
**Jalbani A. et al.**[13]	Pakistan	2009	40	12 - 70	2.6 : 1	AA (32.5%)	HS (22.5%)	MA (15%)	NHL (10%)
**Tariq M. et al.**[14]	Pakistan	2010	50	15 - 70	1.7 : 1	AA (36%)	MA (16%)	MDS (14%)	ALL (12%)
**Santra G. et al.**[15]	India	2010	111	13 - 65	1.5 : 1	AA (22.72%)	HS (15%)	DI (13%)	KA (9%)
**Aziz T. et al.**[16]	Pakistan	2010	88	15 - 60	2.6 : 1	MA (40.9%)	AA (31.9%)	HS and CM (11.4%)	AL (10%)
**Ashraf S. et al.**[17]	Pakistan	2010	150	15 - 60	1.1 : 1	HS (68%)	MA (25.4%)	HM (6.6%)	
**Gayathri B. N. et al.**[18]	India	2011	104	2 - 80	1.2 : 1	MA (74.04%)	AA (18.3%)	Subleukemic leukemia (3.8%)	Malaria (2%)
**Vandana R. et al.**[19]	India	2012	80	1 - 79	1 : 1.2	MA (41.2%)	DA (8.7%)	AA / HA (8.7%)	AL (7.5%)
**Present study**	India		250	All	2.6 : 1	HS (29.2%)	Infections (25.6%)	Myelosuppressants (16.8%)	MA (13.2%)

HS: Hypersplenism; MA: Megaloblastic anemia; AA: Aplastic Anemia; AL: Acute leukemia; HA: Hypoplastic anemia; BM: Bone marrow; DI: Drug induced; KA: Kala-azar; CM: Chronic malaria; HM: Hypoplastic marrow; NHL: Non-Hodgkin’s lymphoma; MDS: Myelodysplastic syndrome; ALL: Acute lymphoblastic leukemia; DA: Dimorphic Anemia.

A definite male predominance observed in our study has been reported by many other similar studies (Table 2), which could be due to social / cultural taboos in our society, making health care facilities more readily available to males as compared to females leading to increased male presentation at hospitals especially in rural areas.

In our study, majority n = 109 (43.6%) cases presented in 3rd and 4th decade (Figure 1) which included maximum number of cases in the major causes of pancytopenia i.e. 35 (47.9%) cases in hypersplenism group, 40 (62.5%) cases in the infections group and 17 (51.5%) cases in megaloblastosis group (Table 3), explaining the finding. Few other studies too have reported 3rd and 4th decade as the commonest age group for presentation of pancytopenia [12,14,15].

**Table 3:** Age distribution of various causes of pancytopenia

**Causes**											
**Age group (in years)**	**Hypersplenism**	**Infections**	**Myelosuppressants**	**Megaloblastosis**	**HA/AA**	**Leukemia**	**Lymphoma**	**PCD**	**MDS**	**Miscellaneous**	**Total**
**Below 1 year**	-	3	-	-	-	-	-	-	-	-	3
**1 – 10**	1	3	5	-	3	3	-	-	-	-	15
**11 – 20**	6	3	2	4	3	1	-	-	-	-	19
**21 – 30**	15	22	3	6	-	1	-	-	-	6	53
**31 – 40**	20	18	2	11	3	-	-	-	-	2	56
**41 – 50**	12	6	6	6	1	2	-	-	-	1	34
**51 – 60**	11	3	12	2	-	-	2	2	1	11	34
**61 - 70**	6	5	8	2	1	-	-	-	-	2	24
**71 – 80**	1	1	4	1	1	-	-	-	-	-	8
**81 - 90**	1	-	-	1	-	-	-	-	-	1	3
**91 - 100**	-	-	-	-	-	-	-	-	-	1	1
**Total**	73	64	42	33	12	7	2	2	1	14	250

The commonest cause of pancytopenia in the present study was hypersplenism (29.2%) whereas in other similar studies the incidence varies from 3 to 68% (Table 2). We think that this may be due to the increasing trend of chronic alcoholism in today’s society; hence more and more patients present with chronic liver disease and decompensated liver cirrhosis, hypersplenism being one of the consequences.

In hypersplenism there is peripheral pooling or trapping and destruction of cells in an enlarged spleen resulting in cytopenias. Increasing severity of the condition causes pancytopenia, as is seen in patients with chronic liver disease and thus hypersplenism may come out to be a common cause for pancytopenia [17,18,20].

Increase in incidence of hypersplenism in our study may also be related to the increased prevalence of malaria, kala azar and other infectious diseases in India, malaria in particular being endemic, especially in the state of Maharashtra. Thus, the incidence of pancytopenia due to hypersplenism caused by parasitic infections is subject to enormous geographical variation. In tropical countries, the incidence is as high as the frequency of splenic enlargement caused by tropical parasitic infections: malaria, leishmaniasis, brucellosis, and schistosomiasis [11].

Malaria, especially Plasmodium falciparum, may cause pancytopenia as a result of hypersplenism, immune hemolysis, DIC, bone marrow necrosis, hemophagocytosis, impairment of marrow function, or direct bone marrow invasion by the parasite. Hamid et al. reported hypersplenism (28%) and malaria (17.3%) to be the two most common causes of pancytopenia contributing for more than 45% of cases in his study of 75 pancytopenic patients [11].

Some of the other studies also found malaria to be a common cause for pancytopenia but did not group malarial infestation under hypersplenism, which is the case in our study [4,5,10,16]. Our findings are consistent with theirs even if malarial infestation as such is considered as a separate cause of pancytopenia without considering the effects of ensuing hypersplenism. All the patients of malaria related pancytopenia in our study made complete hematological and clinical recovery after appropriate treatment.

Though only three (4.2%) cases were diagnosed as Tropical splenomegaly in this group, this figure could be still higher and a major bulk of idiopathic cases may turn out to be tropical splenomegaly, if detailed investigations like measuring malaria antibody levels, seeing for clinical and immunological response to antimalarials etc., are undertaken as outlined by Fankule, as malaria is endemic in India [21].

Infections causing pancytopenia was the 2nd commonest cause of pancytopenia, n = 64 (25.6%) cases in our study. Of these, the majority were patients suffering from AIDS n = 30 (46.9%) cases and septicemia n = 14 (21.9%) cases. HIV infection and overwhelming bacterial infections are known to cause various hematological manifestations including pancytopenia. Savage et al. reported AIDS to be the third most common cause of pancytopenia in his study of 134 hospitalized pancytopenic patients in Zimbabwe [7]. Devi P. M. et al. also found 6% of pancytopenia cases due to HIV infection in her study of 50 cases [12].

Currently 30 – 40 million people are infected with HIV; most of them are in Sub-Saharan Africa and South East Asia. Profound hematological abnormalities are amongst the commonest and maybe the first clinico-hematological manifestations of HIV infection and AIDS, involving all cell lineages of blood. Hematological manifestations are diverse and common throughout the course of HIV infection and maybe attributable to the direct and indirect effects of HIV infection, opportunistic infections and neoplasms, and side effects of therapy. Cytopenias are the most frequent of these abnormalities and the frequency and severity of cytopenias increase in the advanced stages of the disease. Anemia is the most common cytopenia, others being granulocytopenia with or without lymphopenia and thrombocytopenia [22,23].

Of the 14 cases of septicemia due to various infections, two patients showed signs of DIC and one showed evidence of bone marrow necrosis on marrow aspiration. Similar studies showed the rate of pancytopenia due to severe chronic infections to range between 4 – 9% [3,6,8], but the details of the various types of infections was not available for comparison.

We had nine (14%) cases of enteric fever with pancytopenia in this group (Table 1), all of them recovered from the infection uneventfully after appropriate treatment indicating that peripheral blood changes in general and the pancytopenia in specific did not influence the outcome of the disease as suggested by James J et al. [24].

Typhoid fever, which continues to be a major public health problem in countries like India despite the improvements in living standards, has shown changing modes of presentation and complications over the last few years. Though leucopenia with or without neutropenia are characteristic hematological findings; its association with pancytopenia has been reported infrequently in English literature [25]. Development of pancytopenia in enteric fever has been attributed to bone marrow suppression, necrosis, infection associated hemophagocytic syndrome, disseminated intravascular coagulation and development of septicemic complications [26].

All of the eight (12.5%) cases of tuberculosis in this group, had presenting symptoms of fever and weight loss and were put on Anti-Koch’s therapy (AKT), pancytopenic presentation being after a varying duration of 2 – 4 months.

Tuberculosis is a common disease in India and in many other countries. Miliary (disseminated) tuberculosis is known to cause pancytopenia and there are sporadic reports of pulmonary tuberculosis too causing pancytopenia. Although pancytopenia appears to be a rare presentation of tuberculosis, it is advised to always consider tuberculosis as differential diagnosis in patients presenting with pancytopenia, unexplained pyrexia and weight loss. Degree of pancytopenia is influenced more by duration of infection than by its severity [27,28].

The pathogenesis of pancytopenia in tuberculosis has intrigued both physicians and pathologists for years, the exact pathogenesis being not known [28]. Numerous hypotheses have been put forward to explain the occurrence of pancytopenia in tuberculosis and both the tubercle bacilli as well as the AKT have been implicated in its pathogenesis [29]. It has also been suggested by Glasser et al. that other causes of pancytopenia must be diligently sought for in these patients [30]. Other causes of pancytopenia were excluded on clinical and laboratory evaluation in our cases.

All said and done, when tuberculosis occurs with pancytopenia, the mortality rate is high despite of anti-tuberculosis treatment although there are a few reports claiming recovery for their patients [28]. Considering that tuberculosis is a common disease in countries like India, the number of patients who are taking AKT at any particular time and the number of patients who are initiated on AKT in a year, there remains an immense potential for the occurrence of blood dyscrasias including pancytopenia in such patients.

Thus, the physicians should remain alert to the fact that these may be caused by the disease itself or by the drugs when on AKT and so should have a high index of suspicion paving way for early detection and intervention leading to overall reduction in morbidity and mortality in these patients [31].

We also had 3 patients in this group presenting with fever, jaundice and pain in the hypochondriac region with deranged liver function tests, diagnosed to be suffering from viral hepatitis but tests for hepatitis A, B & C virus were negative. CBC reports of these patients showed moderate pancytopenia with normocytic normochromic anemia and normal reticulocyte count.

Pancytopenia with or without aplastic marrow has been reported with increasing frequency in association with variety of viral illnesses, especially infectious hepatitis. Viral hepatitis has been known to cause transient pancytopenia during the course of illness and has also been associated with aplastic anemia, virus of unidentified types being the most common ones. Hepatitis associated pancytopenia and aplastic anemia are usually fatal with a mortality rate as high as 85% [32].

Few other studies too have reported infections like septicemia, enteric fever, tuberculosis, HIV etc. as independent causes of pancytopenia without integrating them together under “infections” group, as is done in our study [3,4,7,9,12,13,15].

Relative to other studies, the overall incidence of infections causing pancytopenia appears to be high in our study. The likely explanation being that our hospital, as a tertiary care centre caters to the poor rural population of southern Maharashtra where the overall health awareness is suboptimal. Thus, patients frequently arrive in hospital after considerable delay, with advanced disease and overwhelming widespread infections, which are difficult to control even with advanced therapy, as is evident from the 42.8% mortality rate in these septicemic patients.

Use of myelosuppressants was the 3rd most common cause for pancytopenia in our study accounting for a total of 42 (16.8%) cases. Patients developing pancytopenia secondary to myelotoxic chemotherapy for hematologic or other malignancies were excluded in few of the studies, causing a selection bias between our and their study [3,5,11,14,16,18]. Santra G. et al. reported 13.5% cases of pancytopenia secondary to chronic use of drugs including chemotherapy in their study of 111 patients, which is comparable to ours [15].

Our institute has separate oncosurgery and radiotherapy units considered as a regional referral centre, more so for carcinoma of breast and oral malignancies which accounted for 18 (42.8%) out of 42 cases in this group. Chemo and radiotherapy cycles are known to cause pancytopenia due to bone marrow suppression, which is considered as an extension of the therapeutic effect, such complications being a part of the natural course of the therapy for the disease and were not an unexpected finding in our study.

Megaloblastic anemia and hypoplastic/aplastic anemia were the 4th and 5th most common causes of pancytopenia, accounting for 33 (13.2%) and 12 (4.8%) cases respectively. The commonest cause of pancytopenia reported in majority of the studies from various parts of the world has been megaloblastic anemia or hypoplastic/aplastic anemia (Table 2), which is in sharp contrast with the results of our study. However, the incidence of megaloblastic anemia and hypoplastic/aplastic anemia in other studies varies from 0.8 to 74% and 7.7 to 52.7% respectively (Table 2) and our findings are still comparable.

Diagnosis of megaloblastosis in this study was established by bone marrow findings and further estimation of folic acid and vitamin B_12_ levels was not performed. The exact cause of deficiency of these vitamins was also not detected, as further tests to determine the etiology are difficult and expensive to perform in most instances. Thus, all the patients in this group were treated with both folic acid and parenteral hydroxycobalamin therapy, with complete documented clinical and hematological recovery in all.

Of the seven (2.8%) cases in leukemia group, four cases were ALL and three were AML (two cases of M3 and one of M1). The incidence of acute/subleukemic leukemia in other similar studies varies from 1.8 to 14.5% (Table 2). Whilst ALL in childhood is the most common association, pancytopenia preceding AML has been described in the literature and adults are occasionally known to be affected [5,8,12,14].

We had 2 (0.8%) cases of NHL with evidence of bone marrow involvement but no splenomegaly and were not on myelosuppressants at the time of pancytopenic presentation. NHL is known to infiltrate bone marrow more commonly than Hodgkin’s disease and thus leading to pancytopenia [2]. The incidence of NHL in other similar studies varies from 0.9 to 10% (Table 2).

We found 2 (0.8%) cases of plasma cell dyscrasia presenting as pancytopenia. Similar other studies too have reported multiple myeloma presenting as pancytopenia, the incidence varying from 0.9 to 4% (Table 2).

We also found a single (0.4%) case of pancytopenia due to MDS of Refractory Anemia with Excessive Blasts (RAEB) type. The incidence of MDS as reported in other similar studies varies from 0 to 18% (Table 2). MDS are most common in the elderly and should be included in the differential diagnosis of elderly patients with pancytopenia, even if mild. Pancytopenic presentation is more common with MDS-RAEB type [9,20].

## Conclusions

Hypersplenism due to decompensated alcoholic liver cirrhosis and infections like HIV and tuberculosis are on rise in today’s society, more so in this part of the world and hence should be kept in mind as causes for pancytopenic presentation.

Anti-neoplastic therapy cycles are known to cause pancytopenia due to myelosuppression and so these hazardous therapies should not be used without facility for regular follow-up and supervision.

Enteric fever, malaria, tuberculosis, megaloblastic anemia are relatively benign, easily treatable and reversible causes of pancytopenia and thus have a good prognosis. Their early diagnosis is important to prevent further complications.

Overwhelming bacterial infections and septicemia as a cause of pancytopenic presentation particularly in the developing countries should always be kept in mind. Early and aggressive treatment initiation should be a priority in these patients, as if left untreated the prognosis is bad.

Detailed clinical history and meticulous physical examination along with baseline hematological investigations provide invaluable information in the evaluation of pancytopenic patients, helping in systematic planning of further investigations to diagnose and ascertain the cause, avoiding unnecessary tests which not only add to the expense of treatment but sometimes also may result in delayed diagnoses and treatment.

As a large proportion of causes for pancytopenia are treatable and reversible, accurate diagnoses and timely intervention maybe lifesaving and will certainly have impact on the morbidity and mortality in these vulnerable patients. Knowing the exact etiology is thus important for specific and timely treatment and for prognostication.

As the etiologies of pancytopenia are varied, so is the prognosis. In our study majority of the cases had a treatable cause and so carried better prognosis.

General physicians who are not hematologists are unlikely to be as well versed in the specific constellation of findings that characterize individual hematologic entities. Stringent diagnostic criteria and a general conceptual framework for ascertaining the cause of pancytopenia is therefore very valuable and a demand of time.

## Competing interests

We declare that we have no sources of support and competing interest to declare.

## Authors’ contributions

AJ designed and performed the study, acquired data, analysed and interpreted the data, and drafted the article. MN conceptualized and designed the study and analysed the data. This study was conducted by AJ while working as Assistant Lecturer, in Krishna Institute of Medical Sciences University (KIMSU), Karad, Maharashtra, India. Both authors read and approved the final manuscript.

## Pre-publication history

The pre-publication history for this paper can be accessed here:

http://www.biomedcentral.com/2052-1839/13/10/prepub
